# Inhibition of corrosion on API 5L X52 pipeline steel in acid media by *Tradescantia spathacea*

**DOI:** 10.3389/fchem.2024.1372292

**Published:** 2024-03-28

**Authors:** Adriana Rodríguez-Torres, María Guadalupe Valladares-Cisneros, German Chávez-Díaz, Víctor Martínez-Calzada, Alonso Saldaña-Heredia

**Affiliations:** ^1^ Metropolitan Polytechnic University of Hidalgo–UPMH Tolcayuca Boulevard, Tolcayuca, Mexico; ^2^ School of Chemical Sciences and Engineering, Autonomous University of Morelos State, Cuernavaca, Morelos, Mexico; ^3^ Research Center for Engineering and Applied Sciences, Autonomous University of Morelos State, Cuernavaca, Morelos, Mexico

**Keywords:** corrosion inhibitor, electrochemical impedance spectroscopy, inhibition efficiency, potentiodynamic polarization curves, weight loss

## Abstract

The concentration effect of *Tradescantia spathacea (T. spathacea)* as corrosion inhibitor of API 5L X52 steel in 0.5 M of H_2_SO_4_ was studied here through electrochemical and gravimetric techniques. To achieve it, samples of the material were prepared to be submitted to each of the tests. Results from electrochemical impedance spectroscopy (EIS) showed that there was an optimum concentration of the inhibitor in which is reached the maximum inhibition efficiency, displaying the best inhibition characteristics for this system with a maximum inhibition of 89% by using 400 ppm. However, the efficiency decreased until 40% when the temperature was increased to 60°C. Potentiodynamic polarization curves (PDP) revealed that some of the present compounds of *T. spathacea* may affect anodic and cathodic process, so it can be classified as a mix-type corrosion inhibitor for API 5L X52 in sulfuric acid. Also, this compound followed an adsorption mechanism; this can be described through a Frumkin isotherm with an adsorption standard free energy difference (ΔG°) of −56.59 kJmol^−1^. Metal surface was studied through scanning electron microscope, results revealed that by adding inhibitor, the metal surface is protected; also, they evidenced low damages compared with the surface with no inhibitor. Finally, *Tradescantia spathacea* inhibited the corrosion process with 82% efficiency.

## Introduction

One of the most used materials in the metallurgic, chemical, and oil industries is mild steel, which can show different types of corrosion depending on the conditions and corrosive media (About et al., 2021). This is a serious problem for the industry as it represents high economic loss and, on occasion, accidents that involve the loss of human life (Mourya et al., 2014; Popoola, 2019). In the oil industry, the high solubility of corrosive gases, such as H_2_S, CO_2_, and O_2_, in the aqueous phase of hydrocarbons contributes to the intensification of the corrosion (Vasquez Medrano et al., 2003). The interaction between O_2_ and H_2_S promotes the formation of elemental sulfur, whose dissolution in water may lead to FeS, Fe_2_O_3_, FeOOH, and S. Likewise, the presence of O_2_ and SO_2_ may result in the formation of sulfuric acid, which increases the corrosiveness of water and induces the formation of FeSO_4_,·4H_2_O, FeOOH, and FeSO_3_xH_2_ scales (Hua et al., 2015; Sun et al., 2016; Likhanova et al., 2018).

Inhibitors are one of the most-used methods of delaying the corrosion process due to their ease of application, availability, and cost effectiveness. These inhibitors include organic, inorganic, and natural products or eco-friendly materials (Finšgar and Jackson, 2014; El-Aouni et al., 2021; Hsissou et al., 2022a).

Inhibitors are substances that are adsorbed on the metal surface, forming a barrier between the metal and the environment that surrounds it; this thin layer results from the physical and chemical attraction between the composite and the metal surface (Touhami et al., 2000; Ozyilmaz et al., 2008). Research about corrosion inhibitors is establishing criteria for appropriate corrosion inhibitor selection. Two distinct moieties can be recognized in the structure of a molecule designed to inhibit corrosion: one or several long hydrocarbons and a “head” that consists of a functional group or a ring system, often containing heteroatoms with electron lone pairs, such as nitrogen, oxygen, and sulfur, to which the chains are attached (Gece, 2008; Álvarez Manzo et al., 2013; Hsissou et al., 2021).

Numerous organic compounds have been studied and applied industrially as corrosion inhibitors. However, in recent years, research in this field has focused on green inhibitors, effective molecules with a low or null environmental impact. Some of the vegetal species extracts used as corrosion inhibitors of steel when sulfuric acid is employed are sunflower-head extract, which presents an efficiency of 92% at a concentration of 3 g/L at 25°C (Wang et al., 2022). *Brassica oleracea L.* obtained an efficiency of 92% at a concentration of 300 mg/L (Li et al., 2021), *Citrus aurantifolia* achieved an efficiency of 96% at a concentration of 250 mg/L (Haldhar et al., 2019), and *Hymenea stigonocarpa* reached 87% at a concentration of 1,233 mg/L (De Britto and Spinelli, 2020). Other extracts have been tested as corrosion inhibitors for mild steel in acidic media.

**FIGURE 1:**
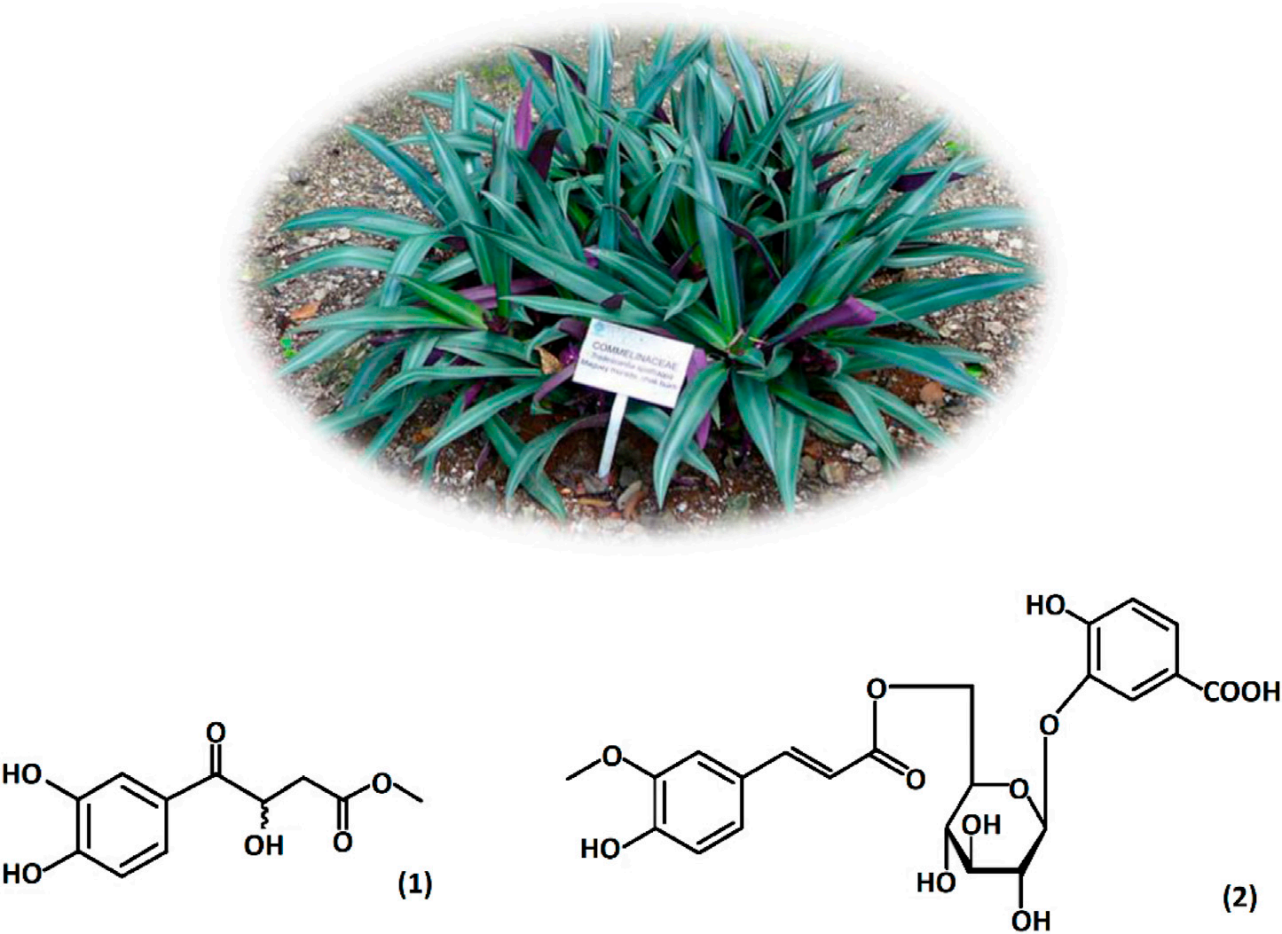
*Tradescantia spathacea* leaves and present compounds: tradescantin (1) and tradescantoside (2). Hudson W. A (2015). Jardín Botánico Dr. Alfredo Barrera Marín, Mexico.

Figure 1 shows *Tradescantia spathacea* which is commonly known as purple maguey; it is an herbaceous species from the Commelinaceae family, native to Mexico and Central America (Hunt et al., 1994). Some studies showed that *Tradescantia spathacea* contains compounds with antioxidant activity, and phytochemical studies have revealed the presence of tannins, terpenoids, alkaloids, glycosides, saponins, and anthocyanins (Rosales Reyes et al., 2008; Tan et al., 2015). Tradescantin (1) and tradescantoside (2) were identified previously in the leaves (Vo et al., 2015), and rhoeonin (cyanidin 3-O-[6-O-(2-O-(feruloyl)-arabinosyl)-glucoside]-7, 3′-di-O-[6-O-(feruloyl)-glucoside]) was the primary anthocyanin identified in the species (Idaka et al., 1987; Tatsuzawa et al., 2010).

The present research shows the results obtained by evaluating *T. spathacea* leaves as a green corrosion inhibitor of API 5L X52 pipeline steel in acidic media. The best inhibitor concentration, efficiency, and temperature effects were determined through gravimetric weight loss and electrochemical techniques (PDP and EIS). A scanning electron microscope was used to analyze the surface morphology changes when the inhibitor was added.

## Experiment

### Obtaining the inhibitor

*T. spathacea* leaves were collected in the north of Cuernavaca, Morelos, Mexico. Leaves were subjected to a drying process at room temperature (25°C ± 2°C) for 4 weeks, and then, they were crushed and immersed in methanol (99%). After this process, they were mashed for 3 days in the absence of light; at the end of the mashing time, the liquid part of the mix was filtered, and the excess solvent was evaporated through a rotary evaporator. This process was followed to obtain the methanolic extract of *T. spathacea.*

### Solution preparation

Analytical grade 98%–99% sulfuric acid and distilled water were used to prepare a corrosive solution of 0.5 M H_2_SO_4_; this solution was used to assess the effects of different concentrations of *T. spathacea* as a corrosion inhibitor of API5L X52 steel.

### Toxicity tests using *Lactuca sativa* seeds

The acute static toxicity test was performed on *Lactuca sativa* seeds, where the phytotoxic effects of *T. spathacea* were evaluated on the growth of seeds and seedlings, following the method established by Sobrero et al. (2004). Seeds of *L. sativa* (fungicide- and pesticide-free) were acquired from the Hortaflor brand. A Whatman filter paper was put into sterilized 10-cm-diameter petri cages. The paper was moistened with 5 mL of solution to test different concentrations of *T. spathacea* (0–400 ppm). A positive control of 0.2 M ZnSO_4_ and a negative control of reconstituted water were used. Twenty *L. sativa* seeds were deposited into each cage; they were hermetically sealed and kept in a dark chamber at a controlled temperature (22°C ± 2°C). After 120 h, cages were opened to count the germinated seeds and measure the radicle and hypocotyl length. Each procedure was done three times to ensure the reproducibility of the results.

### Samples preparation

The samples used were API5L X52 steel with a chemical composition by percentage weight of C 0.21, Mn 1.22, Si 0.24, Cr 0.16, S 0.036, Ti 0.04, Nb < 0.05, Cu 0.19, Al 0.032, Ni 0.14, and Mo 0.06; the remainder was Fe. For weight loss tests, samples were cut into coupons of 2 cm height and 0.635 cm diameter. For electrochemical tests, samples were encapsulated in epoxy commercial resin, leaving an exposed area of 0.317 cm^2^. Before the analysis, samples were sanded with SiC paper from 250- to 600-grain grades.

### Scanning electron microscope

The surface morphology of API5L X52 steel in 0.5 M H_2_SO_4_ was analyzed with a LEO 1450VP scanning electron microscope. Micrographs of the steel in the presence and absence of the inhibitor were obtained.

### Gas chromatography-mass spectrometry analysis

*Tradescantia spathacea*, as a green corrosion inhibitor, was analyzed by gas chromatography using an Agilent 6890 System Plus coupled to an Agilent 5973 Network Mass selective detector. The gas chromatograph was equipped with a silica capillary column (30 m × 0.25 mm, 0.25 mm film thickness). The GC temperature conditions were 45°C–250°C with a temperature gradient of 10°C/min, and the green corrosion inhibitor sample was injected as a solution of 1.0 μL at a 0.02 g/L concentration.

The separated components of the methanol extract of *T. spathacea* were identified by mass spectrometry. The mass index fragmentation of each compound was compared with the mass spectra database N-15598 to identify the compounds (Beale et al., 2018; Kind et al., 2018).

### Weight loss tests

The API5L X52 steel coupons were immersed in 150 mL of 0.5 M H_2_SO_4_ in the absence and presence of different concentrations of *T. spathacea* extract at 25°C, 40°C, and 60°C. This temperature interval was selected because it is used in the industry during chemical pickling (Baddini et al., 2007; Abadeh and Mehdi, 2019). After 24 h, the coupons were removed, and the corrosion product formed on the metal surface was mechanically cleared by scrubbing using a nylon brush under running water following the standard procedure (ASTM Committee G-1 on Corrosion of Metals, 2017; Aslam et al., 2020). Then, each coupon was washed with ethanol, rinsed with acetone, and allowed to dry in the air before being preserved in a desiccator. The final weight of each coupon was recorded. Each test was done three times to calculate the weight loss mean. Corrosion speed $v_{corr}$ (corrosion rate) and the inhibitor efficiency (EIW%) were calculated according to the following Eqs (1) and (2):

$$v_{corr}=\frac{87.6∙W}{\rho At},$$ (1)

where W is the weight loss (*mg*), ρ is density (g.cm^−3^), *A* (*cm* ^*2*^) is the specimen area, and *t* is the exposure time (*h*).

$$IEW\%=\left[1-\frac{v_{corr2}}{v_{corr1}}\right]∙100,$$ (2)

where $v_{corr1}$ and $v_{corr2}$ are the corrosion rates of steel samples obtained by weight loss with and without the inhibitor, respectively.

### Electrochemical measurements

Electrochemical impedance spectroscopy (EIS) and polarization potentiodynamic curve (PDP) tests were carried out using a conventional three-electrode cell. API 5L X52 steel was the working electrode, and an Ag/Cl reference electrode with a Luggi capillary and a graphite counter electrode were used to avoid an Ohmic drop. Measurements were achieved three times using an ACM 2000 GillAC potentiostat instrument. Working electrodes were immersed in the solution for 15 min until they reached the open-circuit potential of the stationary state (EOCP) before each measurement. PDP measurements were performed with a speed rate of 1 mV/s in an interval of ±250 mV from the EOCP. The EIS measurements were executed on the stabilized EOCP with a sinusoidal perturbation signal of 10 mV and a frequency range between 10,000 Hz and 0.5 Hz, recording 50 points per decade.

The IEP% values were calculated from potentiodynamic polarization measurements using Eq. (3):

$$IEP\%=\left[\frac{i_{corr1}-i_{corr2}}{i_{corr1}}\right]x100,$$ (3)

where $i_{corr1\;}$ is the corrosion current without the inhibitor and $i_{corr2}$ is the corrosion current with the inhibitor.

The double-layer capacitance, *C*_*dl*_, was calculated following Eq. (4):

$$C_{dl}=\frac{1}{2\pi f_{\max}R_{ct}},$$ (4)

where $f_{\max}$ is the frequency at which the imaginary component of the impedance is maximum and $R_{ct}$ is the transfer resistance.

From EIS measurements, IE% values were calculated with Eq. (5):

$$IE\%=\left[\frac{R_{ct2}-R_{ct1}}{R_{CT2}}\right]X100,$$ (5)

where $R_{ct1}$ and $R_{ct2}$ are the inhibited and uninhibited charge transfer values, respectively.

## Results and discussion

### Weight loss tests

**FIGURE 2:**
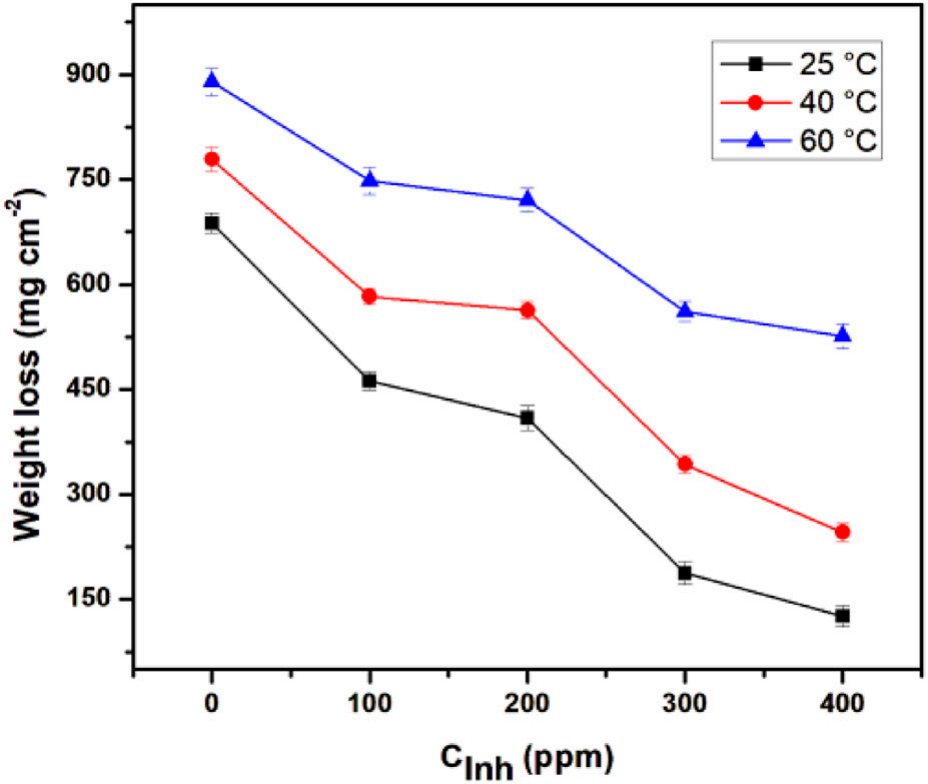
Effect of *Tradescantia spathacea* concentration and temperature on the weight loss of API 5L X52 steel in 0.5 M H_2_SO_4_.

The weight loss measurement is the most accurate and precise method for experimentally determining the metal corrosion rate as it is easy to replicate and, although long exposure times may be involved, the relatively simple procedure reduces the propensity of introducing systematic errors (Zou et al., 2011). Figure 2 shows the effect of increasing temperature in the weight-loss test of API 5L X52 steel with different concentrations of *T. spathacea*.

When the inhibitor concentration was increased, the weight loss was considerably less. However, increased weight loss was observed when the temperature increased; this effect is associated with the decomposition or degradation of the inhibitor.

Results obtained from weight loss measurements are shown in Table 1. The corrosion rate decreased when inhibitor concentrations increased. The best corrosion inhibition efficiency was obtained at 25°C and 400 ppm. However, at 40°C and 60°C, a slight increase in IE% was also appreciated. The IE% behavior was due to a higher amount of *T. spathacea* extract molecules in the corrosive medium; for this reason, a higher number of active sites were protected, reducing the steel mass loss. The protection of the active sites occured because the *T. spathacea* extract molecules possess functional groups and elements with a rich electronic density in their chemical structure (Figure 1), which facilitated the adsorption on the steel surface. The *T. spathacea* extract was more efficient as a corrosion inhibitor at 25°C because its active compounds do not suffer the degradation that possibly occurred at 60°C. At 25°C, the molecule/metal interactions are more stable on the active sites, whereas at 40°C and 60°C, the inhibitor/metal adsorption phenomena are affected by changes in the activation energy promoted by the temperature effect (Hamdy et al., 2013).

**TABLE 1:** Inhibition efficiency percent IE% , weight loss Wl , and corrosion rate $v_{corr}$ for 24 h immersion periods at 25°C, 40°C, and 60°C.

Temperature (°C)	C_inh_ (ppm)	Wl (mg cm^−2^)	σ	V_corr_ (mmpy)	θ	IEW%
**25**	0	687.45	14.08	321.69		
	100	462.17	12.49	216.27	0.33	33
	200	409.36	17.33	191.56	0.40	40
	300	187.20	15.99	87.60	0.73	73
	400	126.22	14.74	59.06	0.82	**82**
**40**	0	779.26	17.25	364.65		0
	100	583.07	10.24	272.85	0.25	25
	200	563.15	12.57	263.52	0.28	28
	300	342.82	13.15	160.42	0.56	56
	400	245.46	12.78	114.87	0.69	69
**60**	0	889.83	19.76	416.39		0
	100	747.81	19.16	349.93	0.16	16
	200	720.90	16.24	337.34	0.19	19
	300	561.85	14.05	262.91	0.37	37
	400	526.76	18.02	246.5	0.41	41

### Adsorption isotherm model analysis

**FIGURE 3:**
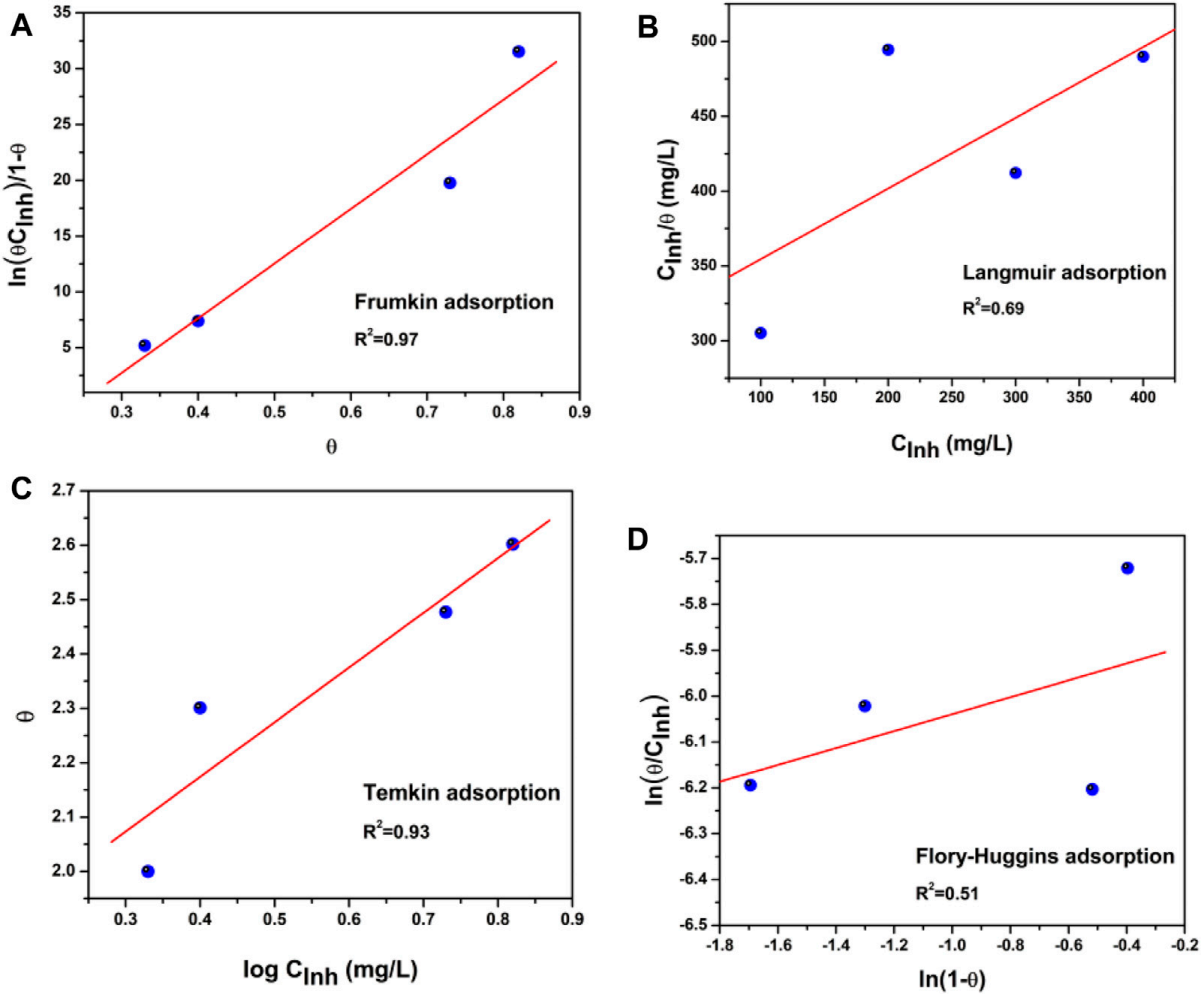
Adsorption isotherms from API 5L X52 steel in 0.5 M H_2_SO_4_ with 400 ppm *Tradescantia spathacea*: **(A)** Frumkin, **(B)** Langmuir, **(C)** Temkin, and **(D)** Flory–Huggins isotherms.

The adsorption process of the corrosion inhibitor is present when it replaces the water molecules and can be adsorbed on the metal surface (Wan et al., 2021; Alao et al., 2022). To further develop the adsorption mechanism, Figure 3 displays different adsorption models to fit the obtained results of the weight loss tests used. The most common are Langmuir, Frumkin, Temkin, and Flory–Huggins isotherms (Abdallah, 2004; Christov and Popova, 2004; Kesavan et al., 2014).

The isotherm that best fitted and described the adsorption behavior of *T. spathacea* was the Frumkin adsorption isotherm, which is represented by Eq. (6)

$$\ln\left[\left(\frac{\theta }{1-\theta }\right)*\frac{1}{C_{inh}}\right]=-\ln{\;K}_{ads}+2\alpha \theta ,$$ (6)

where $K_{ads}$ represents the adsorption at the desorption equilibrium constant, $C_{inh}$ is the concentration of *T. spathacea*, and *α* is the size parameter and a measure of the number of adsorbed water molecules substituted by inhibitor molecules. The covered surface degree *θ* was calculated by using the inhibition efficiency from the weight loss gravimetric technique (IEW%) given by Eq. (7)

$$\theta =\frac{IEW}{100}.$$ (7)

**FIGURE 4:**
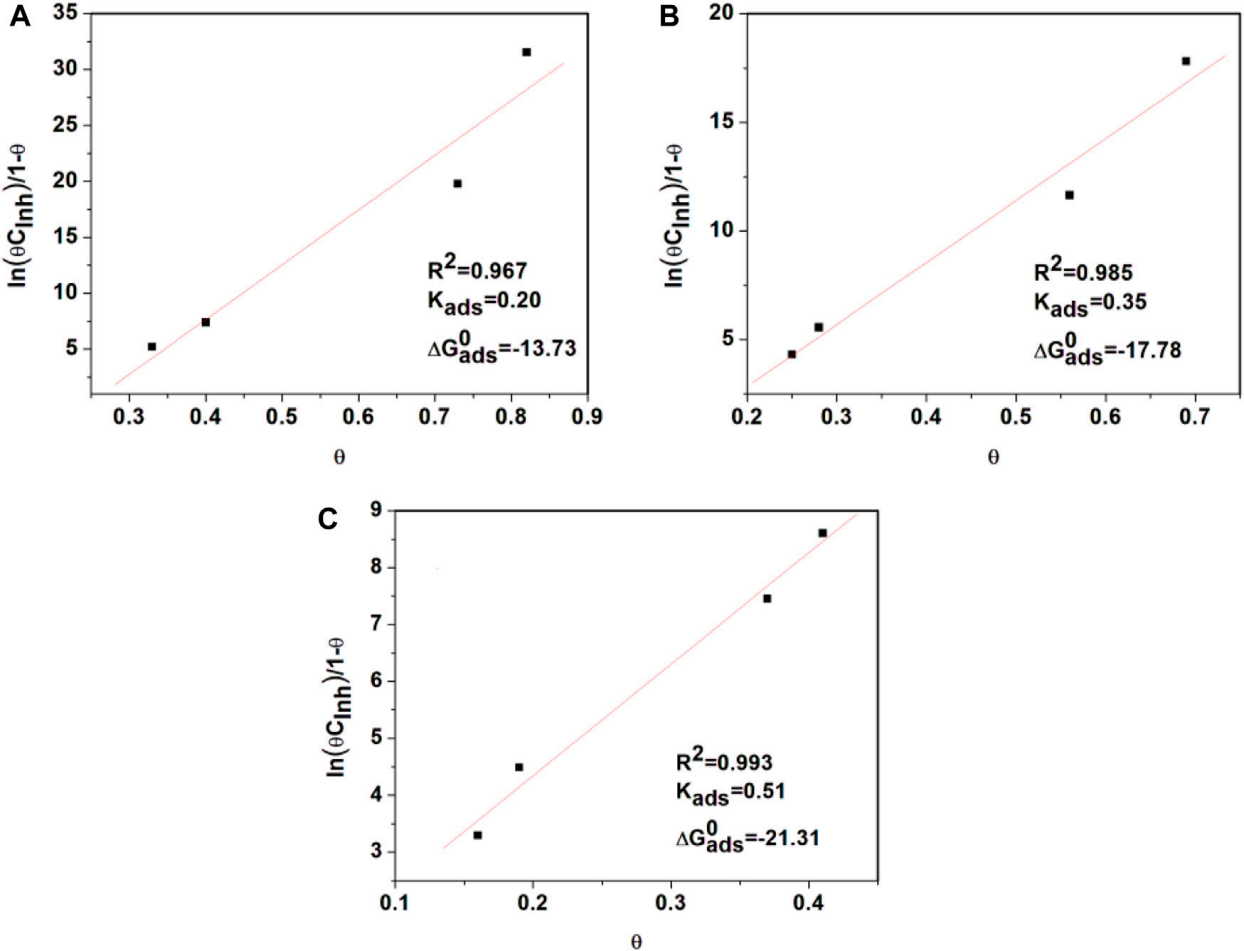
Frumkin adsorption isotherms from API 5L X52 steel in 0.5 M H_2_SO_4_ with 400 ppm *Tradescantia spathacea* at **(A)** 25°C, **(B)** 40°C, and **(C)** 60°C.

In Figure 4, the dependence of the $\ln\ln\;\left(\theta C_{inh}\right)\;/\left(1-\theta \right)$ vs. θ is shown, and the fitting process can be observed. The adsorption coefficient R^2^ of *T. spathacea* at the interface Fe/solution is consistent with the Frumkin adsorption isotherm.

The interaction between the inhibition concentration and the steel surface was evaluated by obtaining the standard free adsorption energy, ${∆G}_{ads}^{0}$ , according to Eq. (8):

$${∆G}_{ads}^{0}=-2.303RT\;\ln\left(C_{H_{2}O}*K\right),$$ (8)

where *R* is the gas constant, $C_{H_{2}O}$ is the concentration of water in the solution expressed in ppm, and *T* is the absolute temperature.

The mechanism of adsorption is physisorption when the value of $∆G_{ads}^{0}$ ≤ −20 kJmol^−1^ and chemisorption when $∆G_{ads}^{0}$ ≥ −40 kJmol^−1^ (Badr, 2009; Benhiba et al., 2021). The obtained values were between −56.59 kJmol^−1^≥ $∆G_{ads}^{0}$ ≤-61.82 kJmol^−1^ at the evaluated temperatures, which indicates that *T. spathacea* has both physical and chemical adsorption onto the steel electrode interface, and chemical adsorption is predominant. *T. spathacea* is mainly chemically adsorbed on the surface of the steel (Tan et al., 2022), showing that the mechanism is physisorption. Additionally, the value of $∆G_{ads}^{0}$ was negative, indicating that the adsorption of the inhibitor on the steel surface can proceed spontaneously (El-Sherif and Badawy, 2011; Ituen et al., 2017).

### Open-circuit potential

The OCP is the potential of a working electrode relative to the reference electrode when there is no current or potential in the cell. The charge in the OCP results in polarization. This occurs as a consequence of the current across the electrode/electrolyte interface (Norsworthy, 2014; Fayomi and Akande, 2019).

**FIGURE 5:**
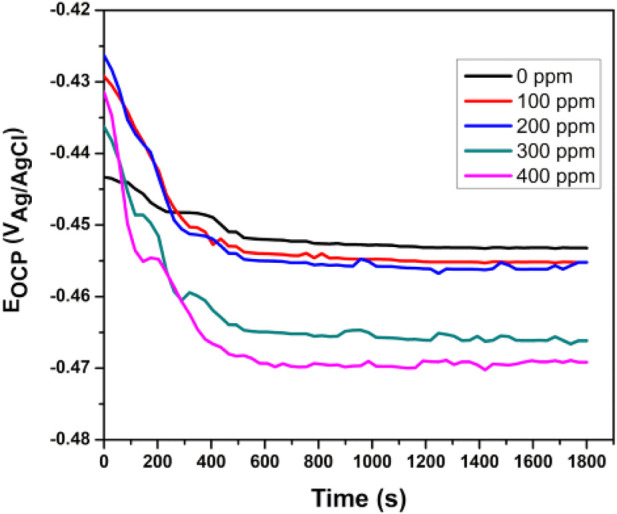
Evolution of open-circuit potential (OCP) versus time for *Tradescantia spathacea* as a green corrosion inhibitor of API 5L X52 steel in 0.5 M H_2_SO_4_ at 25°C.

The stabilized OCP was measured after 1800 s of exposure. Figure 5 shows API5L X52 steel OCP values of 0.5 M H_2_SO_4_ at 25°C with no inhibitor and with different inhibitor concentrations. When the inhibitor was added, the potential was displaced to more negative values. However, it was not as large as −85 mV, and therefore, the inhibitor could not be categorized as a cathodic or anodic type (Hamdy et al., 2013). The stabilized OCP for the blank was −452 mV, while the potential with the inhibitor was between −454 mV and −469 mV. Equilibrium was reached after 600 s in all tests.

### Potentiodynamic polarization curves

**FIGURE 6:**
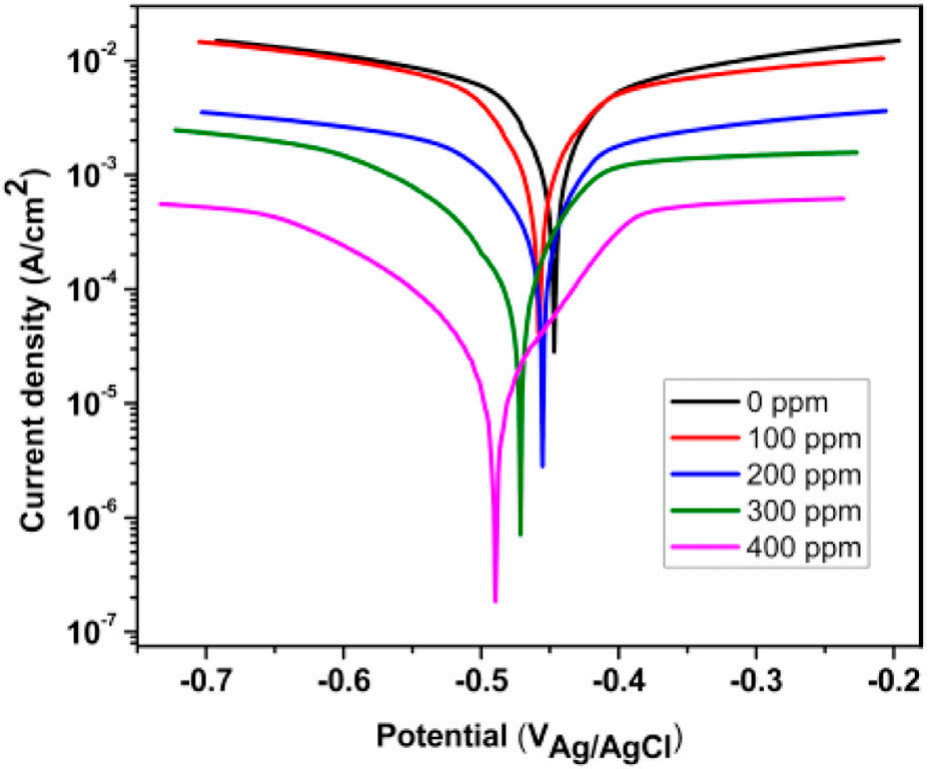
Concentration effect of *Tradescantia spathacea* extract by means of polarization curves for API 5L X52 steel in 0.5 M H_2_SO_4._

Figure 6 shows the PDP results of *T. spathacea* as a corrosion inhibitor of API5L X52 steel in 0.5 M H_2_SO_4_ at 25°C. We observed that the current density was displaced to lower values when the inhibitor concentration increased. Moreover, the potentiodynamic polarization curves were displaced to more negative values with respect to EOCP. This indicates that the inhibitor presence decreased the corrosion speed. This phenomenon may have been caused by the adsorption of *T. spathacea* molecules onto the electrode surface, which changed the concentration of dissolved oxygen at the Fe/solution interface (Tan et al., 2021).

It is evident from Table 2 that the values of β_c_ and β_a_ demonstrated a significant change with inhibitor presence. This change is more evident when the inhibitor concentration increased; the corrosion process drop could be explained by the metal dissolution or as a result of hydrogen evolution on the metal surface because of the adsorption of *T. spathacea* organic compounds on the metal (Alaoui et al., 2016). Compared to the free inhibitor solution, the cathodic and anodic curves of the working electrode in the acid solution containing *T. spathacea* shifted obviously in the direction of current reduction. As was seen from these polarization results, the inhibition efficiency (IEP%) increased with extract concentration, reaching a maximum value of 98% at 400 ppm. In the literature (Ferreira et al., 2004; Singh et al., 2016), it was reported that only when the OCP displacement is at least 85 mV in relation to the displacement measured for the blank solution can a compound be recognized as an anodic or a cathodic inhibitor (Hsissou et al., 2022b). Table 2 indicates that the displacement was at most 44 mV with respect to $E_{corr}$ (the open-circuit potential of the blank solution). Therefore, *T. spathacea* extract might act as a mixed-type inhibitor.

**TABLE 2:** Electrochemical polarization parameters for API 5L X52 steel in 0.5 M H_2_SO_4_ as a function of *Tradescantia spathacea* concentration at 25°C.

Inhibitor [ppm]	-E_corr_ mV	i_corr_ µAcm^−2^	β_a_ mVdec^−1^	-β_c_ mVdec^−1^	IEP%
0	446 ± 0.001	1,017 ± 0.004	13	15	
100	458 ± 0.002	791 ± 0.006	17	13	22
200	455 ± 0.003	398 ± 0.005	44	50	61
300	472 ± 0.002	97 ± 0.002	51	89	90
400	490 ± 0.004	24 ± 0.003	134	200	98

### Electrochemical impedance spectroscopy

**FIGURE 7:**
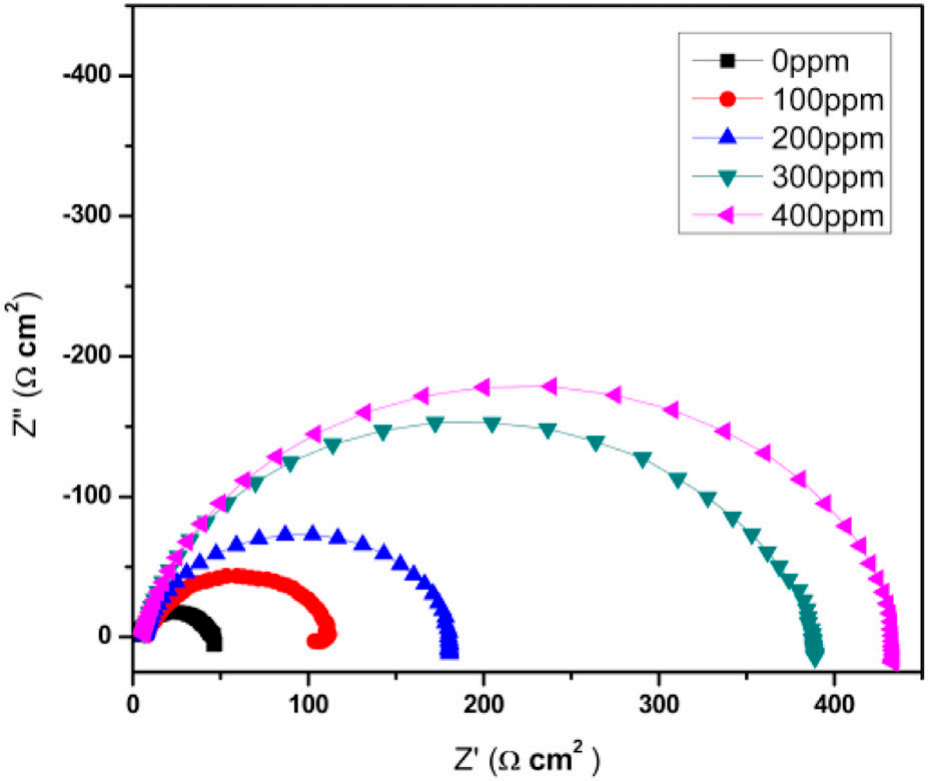
Nyquist diagrams of *Tradescantia spathacea* as a green corrosion inhibitor of API 5L X52 steel in 0.5 M H_2_SO_4_ at 25°C.

Figure 7 shows the Nyquist diagrams for the function of *T. spathacea* concentration as a green corrosion inhibitor of API 5L X52 steel in 0.5 M H_2_SO_4_ at 25°C. For the blank, uninhibited solution, the Nyquist plot described a single and depressed capacitive-like semicircle at all frequency values, with its center on the real axis, indicating that the corrosion process was under charge transfer control from the metal to the electrolyte through the double electrochemical layer. In fact, the semicircle diameter increased, indicating the best corrosion resistance of the protective film.

**FIGURE 8:**
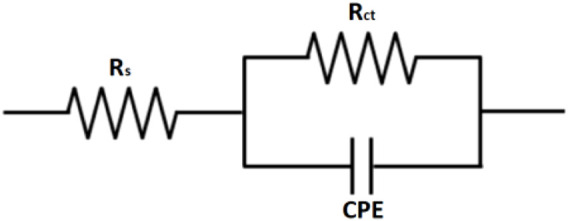
Equivalent circuit used for fitting the obtained data by EIS from API 5L X52 steel in 0.5 M H_2_SO_4_ at 25°C, without and with a corrosion inhibitor.

Figure 8 shows an equivalent circuit model that is proposed to fit and analyze EIS data. The equivalent circuit parameters are presented in Table 3. It can be observed that the value of charge transfer resistance, $R_{ct}$ , is increased by increasing the inhibitor concentration, indicating that the corrosion rate decreased in the presence of the inhibitor, and the inhibition efficiency increased. It is also clear that the value of double-layer capacitance, $C_{dl}$ , decreases when the inhibitor is added, indicating a decrease in the local dielectric constant and/or an increase in the thickness of the double layer structure. This suggests that when the inhibitor concentration is raised, the molecules are adsorbed, forming a protective layer on the metal surface and blocking the active reaction sites available in the metal before they can corrode (Benabdellah et al., 2012). The value of n is between 0 and 1 (0 < *n* < 1). This is related to the deviation from ideal capacitive behavior.

**TABLE 3:** EIS data obtained from API 5L X52 steel in 0.5 M H_2_SO_4_ at 25°C, without and with a corrosion inhibitor.

Inhibitor [ppm]	R_S_ Ω cm^−2^	R_ct_ Ω cm^−2^	C_dl_ µ F cm^−2^	*n*	%IE
0	3	46	61.3	0.91	
100	8	111	38.7	0.87	58
200	8	180	37.4	0.84	74
300	6	388	20.4	0.84	88
400	6	433	13.2	0.83	89

By comparing the results achieved with the different techniques (WL, PDP, and EIS), it is proven that the efficiency of *T. spathacea* natural extract increases with increasing concentration. In the three techniques, the best efficiency percent was reached at a concentration of 400 ppm. PDP efficiency is slightly greater than that reported by the other techniques. This is because The current density causes the ion movement and generates changes in the interface equilibrium state. However, this technique allows for characterizing the corrosion potential and calculating the corrosion current of the metal behavior when an inhibitor is added (Flitt and Schweinsberg, 2005).

### Gas chromatography-mass spectrometry analysis

**FIGURE 9:**
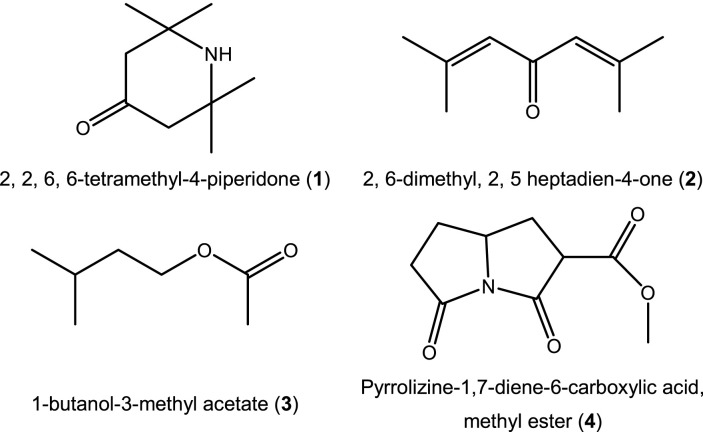
Chemical composition of *T. spathacea* by GC-MS.

GC-MS analysis of the phytoextract from *T. spathacea* used as a green corrosion inhibitor revealed a mixture of different organic and natural components. Table 4 shows the results from GC-MS analysis, and the chemical structures of the compounds identified are displayed in Figure 9.

**TABLE 4:** Chemical components found in *Tradescantia spathacea* by GC-MS analysis.

Compound	Retention time (min)	Amount (%)	Fragmentation index (m/Z)
2, 2, 6, 6-Tetramethyl-4-piperidone (**1**)	7.86	0.4	**155**, 140, 98, 83, 58, and 42
2, 6-Dimethyl, 2, 5 heptadien-4-one (**2**)	8.47	1.01	**138**, 123, 83, and 55
1-Butanol-3-methyl acetate (**3**)	9.78	87.48	**130**, 87, 70, 55, and 43
Pyrrolizine-1, 7-diene-6-carboxylic acid, methyl ester (**4**)	11.53	10.0	**197**, 142, 124, 110, 84, and 44

Four components were separated and identified by GC-MS from the methanol phytoextract of *T. spathacea*. The predominant chemical component in this green corrosion inhibitor was 1-butanol-3-methyl acetate (**3**), which was identified as an essential oil also found in banana peel (Ji and Srzednicki, 2013). The second was pyrrolizine-1,7-diene-6-carboxylic acid, methyl ester (**4**), which has shown anti-insecticidal activity (Hussein et al., 2016). The results of this analysis are different from the literature reports because GS-MS results can vary with aspects such as the geographical location of the plant, the time of harvest, and the extraction method (Qi et al., 2019; Cherif et al., 2021; Ouzir et al., 2021). Compounds 1–3 are considered antioxidants; some authors have related antioxidant active compounds as corrosion inhibitors (Ferreira et al., 2016; Khiya et al., 2019; Laaroussi et al., 2022). However, their corrosion inhibition could be the result of the adsorption of the molecules through their nitrogen and oxygen heteroatoms on the metal surface due to their high electron contribution capacity.

### Corrosion mechanism proposal

**FIGURE 10:**
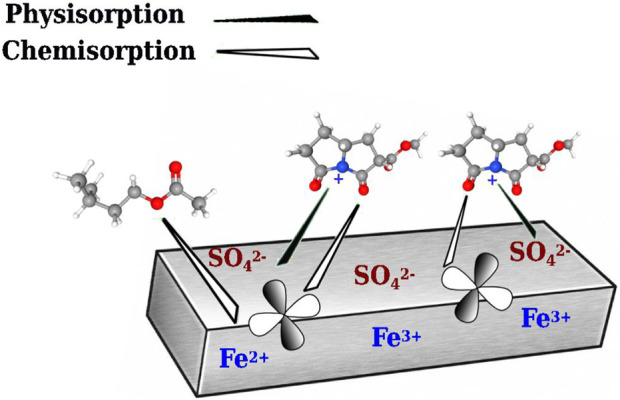
Schematic representation of inhibitor adsorption on the surface of API 5L X52.

The inhibitor has values between −0.674 kJmol^−1^ ≥ $∆G_{ads}^{0}$ ≤ −5.902 kJmol^−1^, indicating that the *T. spathacea* mechanism is physisorption. However, GC results identified two compounds, pyrrolizine-1, 7-diene-6-carboxylic acid methyl ester, and 1-butanol-3-methyl acetate, with a high electron contribution heteroatom capacity, namely, oxygen and nitrogen, which may act as a Lewis base and could form coordinated bonds with the metal’s free *d-*orbitals. This facilitates the adsorption of molecules on the metal surface, forming a protective layer and protecting the metal from aggressive media (Guo et al., 2017). Figure 10 shows a schematic representation of how the inhibitor would interact during chemical and physical adsorption. The chemisorption process provoked a displacement of the adsorbed water molecules on the metal surface and an electron transfer between heteroatoms of pyrrolizine-1,7-diene-6-carboxylic acid methyl ester, 1-butanol-3-methyl acetate molecules, and the metal. The physisorption process is due to the electrostatic interaction of pyrrolizine-1,7-diene-6-carboxylic acid, methyl ester molecules, and ${SO}_{4}^{2-}$ ions. Electrons lost from the iron are donated to the empty orbitals of the extract molecules (Al-Moubaraki and Al-Malwi, 2022). Additionally, the protection effect could be the result of a steric impediment as a consequence of the blockage of adsorbed molecules on the metal surface, preventing the passage of the aggressive media.

### Metallic surface analysis

**FIGURE 11:**
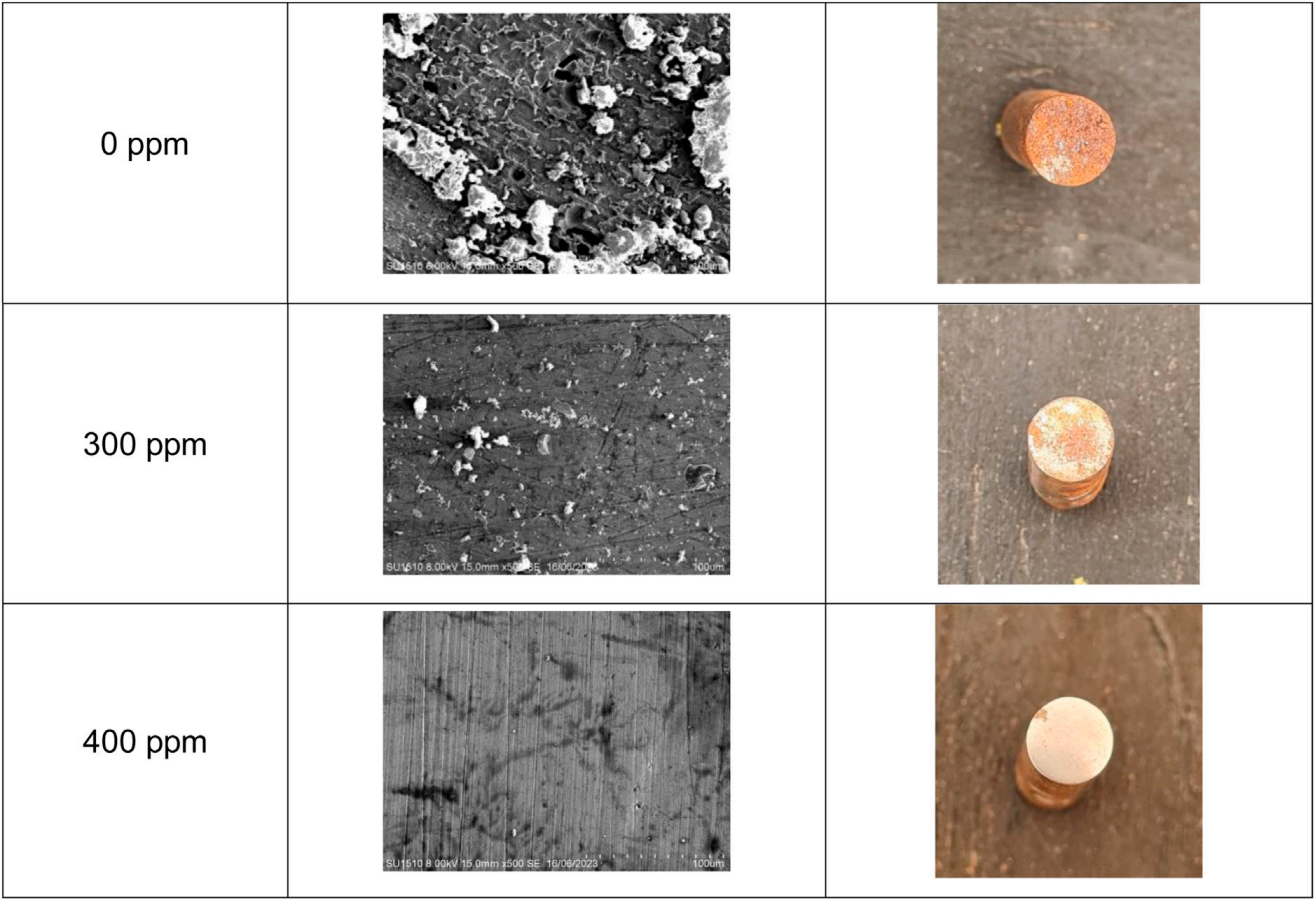
API 5L X52 steel micrographs with 0 ppm, 300 ppm, and 400 ppm of *Tradescantia spathacea* at room temperature.

A surface analysis comparison with 0 ppm, 300 ppm, and 400 ppm of *T. spathacea* at room temperature is shown in Figure 11. It is visible that the metal with no inhibitor revealed more surface deterioration, making the weight loss significant due to iron oxides and other corrosion products. Pitting on the metal surface can be seen. This type of corrosion is one of the most dangerous due to the metal structure, provoking equipment failures (Al-Moubaraki and Al-Malwi, 2022). However, the deterioration decreased when 300 ppm of the inhibitor was added, showing a more protected surface than the one with no inhibitor, and when the concentration was raised to 400 ppm, a homogeneous morphology appeared. This confirmed that an inhibitor decreases the superficial damage and weight loss caused by aggressive media.

## Conclusion

The present work demonstrated that 400 ppm of *T. spathacea* extract is a good corrosion inhibitor of API 5L X52 steel in 0.5 M H_2_SO_4_ at 25°C because this concentration reached an 82% corrosion inhibition efficiency confirmed through the weight loss technique. The PDP demonstrated that the *T. spathacea* extract acts as a mixed-type inhibitor.

The adsorption of *T. spathacea* at the steel interface is consistent with the Frumkin adsorption isotherm; the adsorption of inhibitor molecules onto the steel surface is mainly physical adsorption.

The inhibition efficiency decreases with increasing temperature; even at 60°C, the efficiency remains at 41%.

## Data Availability

The original contributions presented in the study are included in the article/Supplementary material; further inquiries can be directed to the corresponding author.
